# Case Report: A Novel IL2RG Frame-Restoring Rescue Mutation Mimics Early T Cell Engraftment Following Haploidentical Hematopoietic Stem Cell Transplantation in a Patient With X-SCID

**DOI:** 10.3389/fimmu.2021.644687

**Published:** 2021-04-20

**Authors:** Jolanda Steininger, Alexander Leiss-Piller, Christoph B. Geier, Raphael Rossmanith, Reem Elfeky, David Bra, Herbert Pichler, Anita Lawitschka, Natascha Zubarovskaya, Gottfried Artacker, Susanne Matthes-Leodolter, Martha M. Eibl, Hermann M. Wolf

**Affiliations:** ^1^ Immunology Outpatient Clinic, Vienna, Austria; ^2^ Department of Clinical Immunology, Royal Free Hospital, London, United Kingdom; ^3^ Department of Pediatrics, St. Anna Kinderspital and Children’s Cancer Research Institute, Medical University of Vienna, Vienna, Austria; ^4^ Department of Paediatrics and Adolescent Medicine, Danube Hospital, Vienna, Austria; ^5^ Biomedizinische Forschungs GmbH, Vienna, Austria; ^6^ Sigmund Freud Private University- Medical School, Vienna, Austria

**Keywords:** severe combined immunodeficiency, X-SCID, IL2RG, somatic mosaicism, early engraftment, second-site mutation, hematopoietic stem cell transplantation

## Abstract

Mutations of the interleukin 2 receptor γ chain (IL2RG) result in the most common form of severe combined immunodeficiency (SCID), which is characterized by severe and persistent infections starting in early life with an absence of T cells and natural killer cells, normal or elevated B cell counts and hypogammaglobulinemia. SCID is commonly fatal within the first year of life, unless the immune system is reconstituted by hematopoietic stem cell transplantation (HSCT) or gene therapy. We herein describe a male infant with X-linked severe combined immunodeficiency (X-SCID) diagnosed at 5 months of age. Genetic testing revealed a novel C to G missense mutation in exon 1 resulting in a 3’ splice site disruption with premature stop codon and aberrant IL2 receptor signaling. Following the diagnosis of X-SCID, the patient subsequently underwent a TCRαβ/CD19-depleted haploidentical HSCT. Post transplantation the patient presented with early CD8^+^ T cell recovery with the majority of T cells (>99%) being non-donor T cells. Genetic analysis of CD4^+^ and CD8^+^ T cells revealed a spontaneous 14 nucleotide insertion at the mutation site resulting in a novel splice site and restoring the reading frame although defective IL2RG function was still demonstrated. In conclusion, our findings describe a spontaneous second-site mutation in IL2RG as a novel cause of somatic mosaicism and early T cell recovery following haploidentical HSCT.

## Background

Severe combined immunodeficiency (SCID) represents a group of disorders characterized by severe disruption of T cell development and function, which leads to immunodeficiency affecting cellular and humoral immunity. Up to now, mutations in 17 genes that cause SCID have been reported that result in the disruption of lymphocyte development at different steps of lymphoid differentiation (1, 2). X-linked severe combined immunodeficiency (X-SCID) is caused by defects in the gene encoding for the common gamma chain (interleukin 2 receptor gamma) and represents the most common form of SCID (3–5). The common cytokine receptor γ-chain is shared by the receptors for interleukin (IL)-2, IL-4, IL-7, IL-9, IL-15 and IL-21 and abnormalities in cytokine signaling reflect the X-SCID immunophenotype (6, 7). These cytokines are essential in early T cell differentiation as well as NK cell development and B cell function. Accordingly, immunological characteristics for IL2RG-linked X-SCID include the absence of T cells in the peripheral blood and lymphoid organs, with additional NK lymphocytopenia. Although B cell count is normal to elevated, differentiation of mature B cells into memory and antibody-producing plasma cells is impaired leading to a T^-^B^+^NK^-^ SCID immunophenotype with hypogammaglobulinemia (1). Patients with X-SCID manifest with a failure to thrive, chronic diarrhea and severe, potentially life-threatening infections that appear early in life and are caused by opportunistic and non-opportunistic pathogens. Live-attenuated vaccines can result in severe and potentially fatal infections (1, 8). Non-infectious complications may include maternal-fetal graft-versus-host disease due to engraftment of maternal T cells (1, 9, 10). Life expectancy without restoring the immune function is limited, with almost total lethality within the first two years of life (8, 11). Most forms of SCID can be cured if an allogeneic hematopoietic stem cell transplantation (HSCT) is offered as soon as possible. Allogeneic HSCT is a well-established treatment option and is considered the gold standard for all forms of SCID since the first successful bone marrow transplant for an X-SCID patient in 1968 (12). HSCT can potentially re-establish a fully or partially functioning immune system in these patients with very good survival rates (13–17). A North American retrospective multicenter study reported an overall survival rate of IL2RG deficient patients of >80% at 10 years post transplantation (18).

We herein report a patient diagnosed with X-SCID caused by a novel IL2RG mutation resulting in an early splice site disruption and defective protein expression and function. The patient underwent a TCRαβ/CD19-depleted haploidentical HSCT and presented with early CD8^+^ T cell reconstitution, due to the establishment of a somatic mosaicism. At that time, the majority of T cells (>99%) represented autologous cells, mimicking early allogeneic T cell engraftment.

## Case Presentation

**Figure 1:**
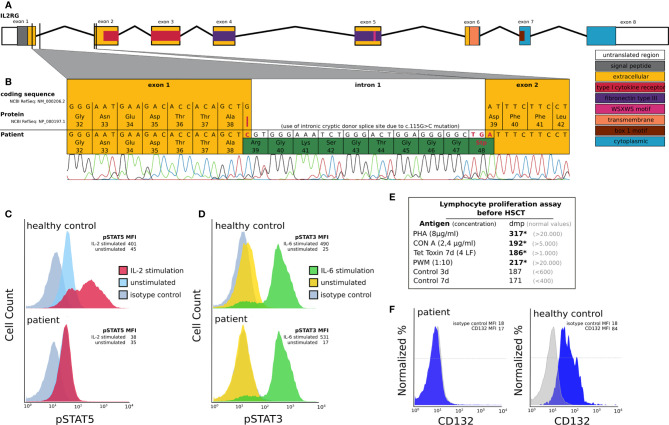
Molecular and functional characterization of a novel IL2RG mutation prior to HSCT: **(A)** Schematic depiction of the IL2RG gene including introns, exons, splice sites and protein domains. **(B)** IL2RG mutation analysis of cDNA derived from EBV transfected lymphoblastoid cell lines of the patient (day -8 pre HSCT). The patient’s IL2RG donor splice site mutation c.115G>C (red line) results in a cryptic donor splice site located at c.115+29 in intron 1. This mutation leads to an insertion of 28 additional nucleotides resulting in a frameshift and premature stop codon at position 48 (highlighted green). **(C, D)** IL-2RG function analysis in an EBV transfected lymphoblastoid cell line of the patient using flow cytometry. We could demonstrate a reduction of pSTAT5 expression following IL-2 stimulation (red curve, C), which is known to be IL2RG dependent, in the patient’s cells compared to those of a healthy control. In C the FACS histograms depicting pSTAT5 expression in IL2-stimulated and unstimulated patient cells overlap. IL2RG independent pSTAT3 expression following IL-6 stimulation (green curve, D) was comparable in patient and healthy control cells. Unstimulated cells are shown in blue (for pSTAT5) and yellow (for pSTAT3). The grey curves depict an isotype control. In D the FACS histograms depicting pSTAT3 expression in unstimulated healthy control cells and the isotype control overlap. The mean fluorescent intensities (MFI) are shown for the patient and the healthy control cells (HC). **(E)** Lymphocyte proliferation assay prior to HSCT. We can detect severely reduced (*) lymphocyte proliferation following stimulation with PHA, CON A, Tetanus toxin and PWM (dmp = disintegrations per minute of 3H-thymidine incorporation). **(F)** IL2RG (CD132) expression on B cells using flow cytometry. The mean fluorescent intensities (MFI) are shown for the isotype control and CD132.

Our patient was the first male child born to non-consanguineous healthy parents of Caucasian ethnicity after an uneventful pregnancy. There was no family history of SCID or other immunodeficiency. At 9 weeks of age he was diagnosed with severe pneumonia and respiratory insufficiency, requiring non-invasive ventilation support. Atypical pneumonia with *Mycoplasma* sp. was suspected due to clinical improvement following treatment with clarithromycin. Later on, the patient also developed nosocomial rotavirus enteritis during hospital admission. Severe hypogammaglobulinemia was detected by routine laboratory assessment. Subsequent extensive immunological workup revealed a T cell lymphopenia, low NK cell counts, a normal B cell count and hypogammaglobulinemia with low IgG and IgA levels (**Table 1**). In addition, lymphocyte proliferation assays revealed a poor response to mitogen (PHA CON A, PWM) as well as tetanus toxin stimulation (**Figure 1E**). Laboratory results were consistent with a T^-^B^+^NK^-^ SCID phenotype. There was no evidence for maternal engraftment. The patient received an antimicrobial prophylaxis consisting of intravenous immunoglobulin (IVIG), fluconazole and sulfamethoxazole/trimethoprim sulfate, upon which no further infections occurred. PCR testing for CMV and other herpesviruses was negative. Due to CMV seropositivity of the mother, the patient was weaned off breastfeeding and put on CMV prophylaxis with ganciclovir. To determine the underlying disease-causing genetic defect we screened for mutations in the primary immunodeficiency genes listed in the 2011 IUIS expert committee report (21). Targeted next generation sequencing revealed an IL2RG c.115G>C (ENST00000374202.2) mutation which was confirmed by Sanger sequencing. This novel IL2RG mutation disrupts the donor splice site of exon 1, as could be demonstrated by cDNA library derived from an EBV transfected B cell line of the patient before HSCT (**Figures 1A, B**). A cryptic donor site at c.115+29 in intron 1 was used, leading to an insertion of 28 intronic nucleotides to the transcript causing a frameshift and premature stop codon at position 48, thus predicting to truncate IL2RG protein (p.Asp39ArgfsTer9). Molecular segregation analysis showed perfect segregation, with the patient’s mother harboring the same missense variant on one allele (data not shown).

**Table 1:** Laboratory parameters of our X-SCID patient prior to and after hematopoietic stem cell transplantation (HSCT) compared to an age matched healthy control cohort. *(19)**(20).

	Age-matched reference (6-12 months)	pre-HSCT	35 days post	42 days post	70 days post	91 days post	126 days post	198 days post	1 year post	1,9 years post	2,6 years post
			HSCT	HSCT	HSCT	HSCT	HSCT	HSCT	HSCT	HSCT	HSCT
Age of the patient at time of analysis		5 months	7,5 months	8 months	9 months	9,5 months	11 months	13 months	18,5 months	29,5 months	37,5 months
lymphocytes (cells/μL)	3400-9000*		1100	1400	1800	2700	6600	7000	9400	10600	10200
CD3^+^/μL	1900-5900*	65	264	524	660	1580	3619	3490	4050	3555	2192
CD4^+^/μL	1460-4300*	30	25	42	40	225	1317	1840	1875	1295	852
Chimerism (CD4^+^)^Ω^			<1%	<1%	23%	82%	94%	98%	99%	98%	98%
CD4^+^CD45RA^+^ T cells (% of total CD4)	64-93*	0	0	0	16	50	45	54	70	55	46
CD8^+^/μL	500-1700*	5	237	483	445	870	1759	1325	1660	1350	707
Chimerism (CD8^+^)^Ω^			<1%	<1%	<1%	39%	41%	77%	93%	91%	86%
CD8^+^CD45RA^+^ T cells (% of total CD8^+^cells)	75-97*	0	0	0	<1	3	16	47	70	62	57
NK cells/μL	160-950*	15	33	7,3	7	5	11	10	12	8	10
Chimerism (NK cells)^Ω^			92%	81%	73%		45%	52%			
CD19^+^/μL	601-2600*	1375	0	0	0	<1	675	865	945	955	578
Chimerism (CD19^+^)^Ω^							<1%	3%	<1%	<1%	
Monocytes/μL	250-1200**	1070	320	309	440	230	429	395	590	800	1053
Chimerism (Monocytes)^Ω^			<1%	<1%	4%	2%	1%	1%	<1%	<1%	<1%
Granulocytes/μL	1100-5600**	6035	450	513	670	800	1666	2065	3745	5015	5998
Chimerism (Granulocytes)^Ω^			<1%	<1%	1%	1%	1%	1%	<1%	1%	2%

Ω represents percentage of donor engraftment.

Expression of IL2RG protein on the surface of the patients EBV-transformed B cells was severely reduced (**Figure 1F**). IL-2 receptor function analysis in EBV transfected lymphoblastoid cell line of the patient was performed using an inhouse intracellular flow cytometry protocol prior to HSCT and confirmed the functional disruption of the common gamma chain. We could demonstrate a significant reduction of phosphorylated signal transducer and activator of transcription (pSTAT5) following IL-2 stimulation of the patient compared to those of a healthy control (**Figure 1C**). STAT3 phosphorylation following IL-6 stimulation, which is known to be common gamma chain independent, was comparable between patient and healthy control cells (**Figure 1D**).

An urgent allogeneic HSCT was planned for the patient, using the CMV seronegative father as the haploidentical donor, as no HLA-matched unrelated donor could be identified. The preparation regimen consisted of rabbit antithymocyte-globulin (rATG) at a dose of 2.5 mg/kg/d (administered on days -5 until -3) and Rituximab 375 mg/m^2^ on day -1. At the age of 28 weeks, the patient received a TCRαβ/CD19-depleted peripheral blood stem cell transplantation, including 23 x 10^6^/kg CD34^+^ cells and <0,01 x 10^6^/kg TCRαβ cells. Prophylaxis against graft-versus-host disease (GvHD) included Ciclosporin A, started on day -1, at 3mg/kg/d in combination with Mycophenolate mofetil (MMF), started on day +1, at 45mg/kg/d until day +27. The patient was discharged in excellent physical condition 12 weeks after HSCT and remained on prophylactic Fluconazole, Cotrimoxazole and Aciclovir as well as immunoglobulin replacement therapy. Routine post-HSCT screening included flow-cytometric analysis of leukocyte engraftment and chimerism analysis from sorted subpopulations performed once weekly (**Table 1**) and weekly PCR testing for CMV, EBV and human Adenovirus.

**Figure 2:**
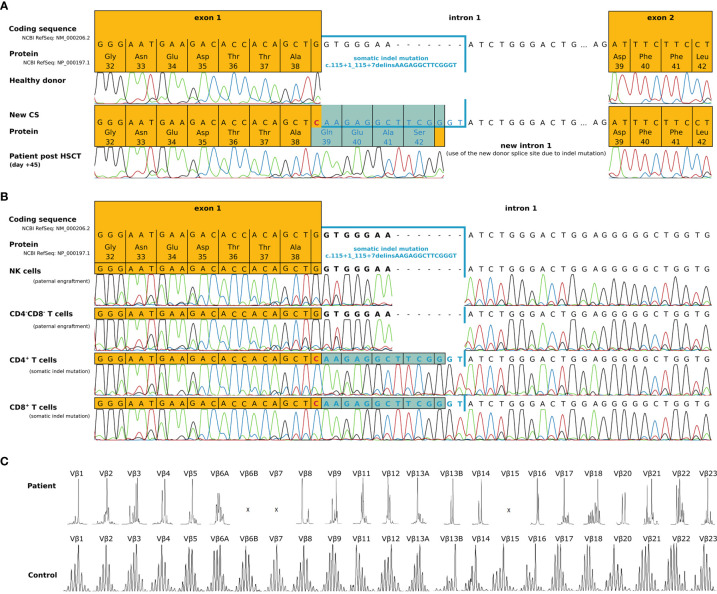
Molecular characterization of a second-site IL2RG mutation 45 days post-HSCT. **(A)** IL2RG mutation analysis of cDNA derived from EBV transfected lymphoblastoid cell lines of the patient (day +45 post-HSCT). Due to a somatic indel mutation in intron 1 (c.115+1_115+7delinsAAGAGGCTTCGGGT), a new cryptic donor splice site within the inserted sequence was created. The transcript showed an insertion of 12 nucleotides, which lead to an inframe insertion of 4 residues (highlighted in blue), restoring the original reading frame. **(B)** Sequencing of genomic DNA from sorted cell populations (NK cells, CD4^-^CD8^-^ double negative T cells, CD4^+^ and CD8^+^ T cells) of the patient (day +45 post HSCT). Paternal engraftment with wild type IL2RG was found within NK cells and CD4^-^CD8^-^ double negative T cells. CD4+ and CD8+ T cells carried the somatic indel mutation c.115+1_115+7delinsAAGAGGCTTCGGGT that restored the reading frame (highlighted blue). **(C)** The distribution of TCR Vβ elements 1-23 in the patient and an age matched healthy control.

**Figure 3:**
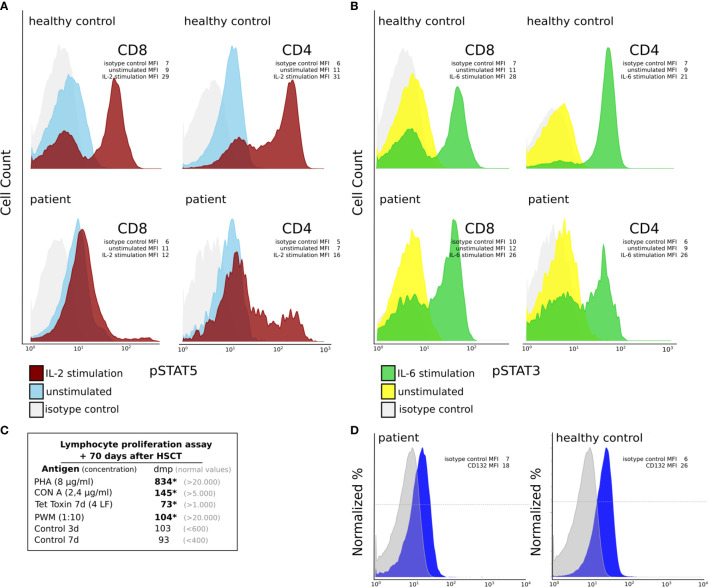
Functional characterization of CD4^+^ and CD8^+^ T cells at day +70 post HSCT. **(A, B)** IL-2RG function analysis in CD4^+^ and CD8^+^ T cells of the patient using flow cytometry. **(A)** We could find decreased pSTAT5 expression following IL-2 stimulation (red curve), which is known to be IL2RG dependent, in CD8+ T cells of the patient compared to those of a healthy control. In the patient pSTAT5 expression could be detected in a small subset of CD4^+^ T cells only. The mean fluorescent intensities (MFI) are shown for the isotype control, IL-2 stimulated and unstimulated cells **(B)** IL2RG independent pSTAT3 expression following IL-6 stimulation (green curve) in CD8+ and CD4^+^ T cells was comparable between patient and healthy control. Unstimulated cell’s expression histograms are shown in blue (for pSTAT5) and yellow (for pSTAT3). The grey curves depict an isotype control. The mean fluorescent intensities (MFI) are shown for the isotype control, IL-6 stimulated and unstimulated cells. **(C)** Lymphocyte proliferation assays +70 days post-HSCT. We can detect reduced (*) lymphocyte proliferation following stimulation with PHA, CON A, Tetanus toxin and PWM (dmp = disintegrations per minute of 3H-thymidine incorporation). **(D)** IL2RG (CD132) expression on T cells using flow cytometry. The mean fluorescent intensities (MFI) are shown for the isotype control and CD132.

In terms of immune reconstitution, CD8^+^ T cells started to increase on day +35 and reached almost normal cell counts on day +42 (**Table 1**). While CD4^+^ T cells were detected as early as day +35 they remained low until day +91. Notably, the flow cytometry and chimerism analyses showed an expansion of autologous CD8^+^ T cells with less than 1% donor chimerism until day +70 post-HSCT while CD4 chimerism analysis revealed a donor chimerism of 23% at day +70 (**Table 1**). Sanger sequencing of the IL2RG region in a cDNA (complementary DNA) library derived from PBMC of the patient on day +45 (**Figure 2A**), as well as in gDNA (genomic DNA) libraries of sorted lymphocytes (1x10^6^ cells/625µl in total), CD4^+^ (5x10^3^), CD8^+^(6x10^4^), CD4^-^ CD8^-^ double negative T cells (1.7x10^3^) and NK cells (2x10^3^) was repeated (**Figure 2B**). Sequencing of B cells at this time was not possible due to low B cell count. We could detect a second-site, spontaneous somatic indel mutation (c.115+1_115+7delinsAAGAGGCTTCGGGT) in the CD4^+^ and CD8^+^ T cells of the patient. This second-site mutation restored the reading frame by introducing a cryptic donor site within the inserted sequence, adding 4 new amino acid residues (GlnGluAlaSer) at position 39 to the protein (p.Ala38_Asp39insGlnGluAlaSer). Sanger sequencing revealed paternal engraftment of NK cells and CD4^-^ CD8^-^ double negative T cells only. CD4^+^ and CD8^+^ T cells carried the above described second-site mutation. T cell receptor diversity was analyzed by complementarity-determining region–3 (CDR3) spectratyping of 23 TCR Vβ elements in patient’s T cells 46 days after HSCT. The majority of the Vβ elements analyzed showed an oligoclonal skewing. The analysis of TCR Vβ elements 1-23 revealed the presence of single peaks, demonstrating the existence of predominant clones expressing these particular Vβ chains (**Figure 2C**). This second-site mutation in CD4^+^ and CD8^+^ T cells did not lead to restoration of IL2RG function. IL-2 receptor function analysis in CD4^+^ and CD8^+^ T cells was performed using flow-cytometry on day +70 post HSCT and showed functional disruption of the common gamma chain comparable to the findings before HSCT. We could demonstrate a significant reduction of pSTAT5 following IL-2 stimulation in CD8^+^ T cells. In only a small subset of CD4^+^ T cells phosphorylation of STAT5 was shown corresponding to the fraction of donor cells within the CD4 population at that time while concomitantly 99% of the CD8 cells were of recipient origin (**Figure 3A** and **Table 1**). STAT3 phosphorylation following IL-6 stimulation, which is known to be common gamma chain independent, was reexamined in parallel and comparable between patient and healthy control (**Figure 3B**). Lymphocyte proliferation assays of PBMC’s tested in parallel are shown in **Figure 3C** and confirm the impaired functionality of the expanded T cell populations. Nevertheless, the second-site mutation enabled partial restoration of IL2RG expression on the patient’s T cells as examined by FACS (**Figure 3D**).

Ninety-one days post HSCT donor CD8^+^ T cell engraftment increased to 39% and reached 93% at one year after HSCT. CD4^+^ T cell count increased to reach 225 cells/µl on day +91 days in parallel with increased CD4 chimerism. CD4 counts were normalized at day +126 post-HSCT. Circulating naïve CD4^+^ and naïve CD8^+^ T cells (CD4^+^CD45RA^+^ T cells and CD8^+^CD45RA^+^ T cells) were detectable at day +71 and day +91, respectively. By day +126 the percentage of naïve CD4 and naïve CD8 reached 45% and 16%, indicating thymic immune recovery (**Table 1**). NK cell lineage showed roughly 90% donor engraftment after 35 days post-HSCT, whereas NK cell count did not recover during this time period and remained low in the follow-up examinations. Autologous B cell recovery was first confirmed at day +113 and reached 200 cells/μL by day +126. As expected without chemo-conditioning, no donor-derived myeloid engraftment was detectable (**Table 1**). The immunoglobulin levels remained low (IgG; IgA; IgM), so that immunoglobulin replacement therapy was continued. During follow-up, the patient remained free from infections and did not develop acute or chronic GvHD.

## Discussion and Conclusions

X-SCID is an immunodeficiency almost universally fatal within the first two years of life unless immune reconstitution is achieved through stem cell transplantation or gene therapy (17, 22). During the last years, patients with atypical clinical presentations or milder phenotypes of X-SCID have been described (23–29), revealing somatic mosaicism with reversions or second site rescue mutations (partially) restoring IL2RG function as detected in some cases (26, 27, 30–32).

We herein describe a young boy with a spontaneous frame-restoring second-site mutation that resulted in a somatic mosaicism and oligoclonal CD8^+^ T cell expansion after HSCT, mimicking early T cell engraftment. Somatic mosaicism in CD4+ and CD8+ T cells of patients with X-SCID was first described in 1996 and can present with variable clinical phenotypes ranging from mild T cell lymphopenia to patients resembling predominantly antibody deficiency (26–28, 30, 31). Similarly, in other primary immunodeficiency disorders reversions have been linked to atypical and relatively milder disease courses including adenosine deaminase (ADA)-deficient SCID (33–37), X-linked ectodermal dysplasia and immunodeficiency (38), Wiskott-Aldrich syndrome (39), JAK3-SCID (40) and RAG1 deficiency (41).

HSCT utilizes three stem cell sources, bone marrow (BM), peripheral blood (PB) and cord blood (CB). The choice of stem cell depends on donor accessibility, disease and urgency for the transplant (42). Immune reconstitution after HSCT in SCID patients is a multifactorial process dependent on stem cell source and manipulation of the graft among other donor and host factors (43–45). It has been shown, that in particular *ex-vivo* T cell depletion and the use of rATG have a significant impact on T cell immune reconstitution in patients transplanted for malignant diseases (46–49). However, the effect of serotherapy on T cell recovery in PID patients seems less clear. Lymphocyte recovery after serotherapy with Alemtuzumab was shown to be dose dependent (50) and rapid expansion of naïve CD4^+^ cells has been described after unrelated cord blood transplantation without serotherapy in a mixed cohort of malignant and non-malignant diseases including PID (51). The decision whether or not to use a conditioning regimen additionally affects immune reconstitution after HSCT for PID. Patients with X-SCID usually develop sufficient donor T cell engraftment after unconditioned HSCT from HLA-matched siblings or T cell depleted haploidentical donors (52) but frequently will remain of host origin in the B cell compartment due to insufficient engraftment of donor-derived stem cells (4). Thus maintaining a status of intrinsically impaired B cell function, such patients often need life-long immunoglobulin substitution. Whether a modern reduced-toxicity conditioning will enable a sustained donor-derived B cell engraftment and finally improve survival in X-SCID patients as suggested by recent data remains to be confirmed (53).

T cell recovery occurs through two distinct pathways; a thymic independent and thymic dependent T cell pathway (54). While thymic-independent T cell reconstitution, by adoptively transferring mature memory/effector T cells is associated with an early T cell reconstitution of 1-2 months, it can result in potentially fatal graft-versus-host disease (GvHD) (44). Modern haploidentical HSCT utilizes *ex-vivo* T cell (αβ) depletion to prevent development of GvHD (42). With limited numbers of donor CD4^+^ and CD8^+^ T cells infused, this type of graft manipulation doesn’t support a thymic independent T cell expansion. Recent studies showed evidence of thymic dependent reconstitution as early as 4-5 months with the use of TCR/CD19 depleted grafts (55, 56).

In addition, rATG as part of the GvHD preparative protocol delays thymopoiesis, with a median time to reconstitute CD4 counts and CD8 counts to ≥200 cells/ul of 10.9 months and 18.2 months, respectively (57).

The patient described herein received a haploidentical HSCT using TCRαβ/CD19-depleted peripheral blood stem cells from his father after preparation with rATG. Additionally, the impaired function of host B cells in X-SCID patients prompted us to administer Rituximab in an attempt to minimize the risk for EBV-associated lymphoproliferative disease (58). Establishment of robust CD8^+^ T cell reconstitution on day 45 in our patient was particularly early after TCRαβ/CD19 depleted HSCT and serotherapy with rATG. Concomitant low numbers of naïve CD45RA^+^CD8^+^ T cells at the same time pinpointed to an oligoclonal expansion of peripheral CD8^+^ T cells. Similar reconstitution dynamics are seen in HSCT utilizing unmanipulated stem cell grafts, where an oligoclonal donor CD8^+^ T cells expansion was noted during the first 6 months post-HSCT (44). However, in our patient, the early oligoclonal CD8^+^ T cell expansion was of autologous origin. A subsequent mutationanalysis revealed a frame-restoring second-site mutation in autologous CD8^+^ T cell and CD4^+^ T cells. Subsequent functional testing in expanded CD8^+^ T cells showed unchanged common gamma chain dysfunction. However, IL2RG protein expression on the patient’s T cells was at least partially restored, indicating that the second-site mutation enabled reduced expression of IL2RG protein with abnormal functionality. Later on, the initially observed oligoclonal expansion of autologous CD8^+^ T cells gradually converted into a stable full donor chimerism due to expansion of donor-derived CD8^+^ T cells including naïve CD8^+^ cells, which were first detected around day +91, indicating a thymic-dependent T cell reconstitution.

We speculate, that the continuous replacement of the expanded, non-functional host CD8^+^ T cells resulted from a selection advantage of the functional CD8^+^ T-cells in common gamma chain signaling.

Functional testing in CD4^+^ T cells on the other hand showed common gamma chain function in a small number of the cells. At the time of analysis (day +70 post HSCT) donor chimerism in the CD4^+^ T cell population had increased to 23%. We therefore could attribute the restored common gamma chain function to the small portion of donor CD4 cells.

CD4^+^ T cell regeneration is highly thymic dependent resulting in a prolonged recovery (59). The rapid expansion of the second-site mutated T cells in our patient might have also supported early CD4^+^ count recovery (>200 cells/μl) and appearance of naïve T cells by day +91 post-HSCT, which is faster as compared to the data by Shah et al, 2018 where sustained CD4^+^ recovery above 200 cells/μl was not achieved before day +129 post-transplant (60).

The appearance of the second-site mutation in mature CD4^+^ and CD8^+^ T cell lines in the absence of the mutation in progenitor cells (double negative T cells) as well as NK cells, suggests that the mutation appeared at a late stage of T cell differentiation, e.g. between double positive T cells to CD8^+^ and CD4^+^ T cells. We were unable to perform sequencing of B cells due to B cell depletion prior to HSCT and absent B cell recovery at that time.

In conclusion, we herein describe a case of a young male infant with a spontaneous frame-restoring second-site mutation in IL2RG that resulted in a somatic mosaicism after allogeneic HSCT. While reversion or second site rescue mutations are known to lead to a variable clinical phenotype of X-SCID, to our knowledge, an oligoclonal CD8^+^ T cell expansion due to somatic mosaicism after HSCT mimicking early T cell engraftment has never been reported so far.

Our report might lead to increased awareness for the differentiation between early oligoclonal expression of autologous non-functional T cells versus thymus-dependent recovery of engrafted functional donor cells.

## Data Availability Statement 

The raw data supporting the conclusions of this article will be made available by the authors, without undue reservation. Requests to access these datasets should be directed to hermann.wolf@itk.at.

## Ethics Statement 

The study was conducted in accordance with the Declaration of Helsinki and fulfills the guidelines of the Austrian Agency of Research Integrity (OeAWI). With respect to the genetic and molecular clinical analyses this study was approved by the Ethics Committee of the Immunology Outpatient Clinic as a study using the residual specimens biobank of the Immunology Outpatient Clinic. According to the Ethics Committee of the City of Vienna and the legal regulations to be applied (§15a Abs. 3a Wiener Krankenanstaltengesetz) no additional ethics committee evaluation is required for a non-interventional study using data collected as part of the routine medical care the patients received. The patient’s parents gave their informed consent that anonymized data collected as part of the routine medical attendance (immunological analysis, flow cytometry analysis and genetic mutation analysis) could be included in a scientific publication. All patient information in this study is anonymized and de-identified. No extra intervention was carried out.

## Author Contributions

JS and CG analyzed and interpreted the results, created the figures and tables, and actively wrote the manuscript. AL-P did the genetic testing evaluation and created the associated figures. RR and DB conducted and interpreted measurements of functional assays in T cells. Critical revision of the article was done by RE, HP, and HW. ME interpreted and analyzed results. The following clinicians HP, AL, NZ, GA, SM-L, and HW cared for the patients and were actively involved in our research investigation. HW took overall responsibility for the research performed in this study and guided the writing of the manuscript. All authors contributed to the article and approved the submitted version.

## Conflict of Interest

Author ME was employed by company Biomedizinische Forschungs GmbH.

The remaining authors declare that the research was conducted in the absence of any commercial or financial relationships that could be construed as a potential conflict of interest.
